# Assessment of Solid Pulmonary Nodules or Masses Using Zero Echo Time MR Lung Imaging: A Prospective Head-to-Head Comparison With CT

**DOI:** 10.3389/fonc.2022.812014

**Published:** 2022-04-26

**Authors:** Qianyun Liu, Zhichao Feng, Weiyin Vivian Liu, Weidong Fu, Lei He, Xiaosan Cheng, Zhongliang Mao, Wenming Zhou

**Affiliations:** ^1^ Department of Medical Imaging, Yueyang Central Hospital, Yueyang, China; ^2^ Department of Radiology, The Third Xiangya Hospital, Central South University, Changsha, China; ^3^ Magnetic Resonance (MR) Research, General Electric (GE) Healthcare, Beijing, China

**Keywords:** magnetic resonance imaging, zero echo time sequence, pulmonary nodule, comparison, computed tomography

## Abstract

**Objective:**

The aim of this study is to determine the potential of zero echo time (ZTE) MR lung imaging in the assessment of solid pulmonary nodules or masses and diagnostic consistency to CT in terms of morphologic characterization.

**Methods:**

Our Institutional Review Board approved this prospective study. Seventy-one patients with solid pulmonary nodules or masses larger than 1 cm in diameter confirmed by chest CT were enrolled and underwent further lung ZTE-MRI scans within 7 days. ZTE-MRI and CT images were compared in terms of image quality and imaging features. Unidimensional diameter and three-dimensional volume measurements on both modalities were manually measured and compared using the Wilcoxon signed-rank test, intraclass correlation coefficient (ICC), Pearson’s correlation analysis, and Bland–Altman analysis. Multivariable logistic regression analysis was used to identify the factors associated with significant inter-modality variation of volume.

**Results:**

Fifty-four of 71 (76.1%) patients were diagnosed with lung cancer. Subjective image quality was superior in CT compared with ZTE-MRI (*p* < 0.001). Inter-modality agreement for the imaging features was moderate for emphysema (kappa = 0.50), substantial for fibrosis (kappa = 0.76), and almost perfect (kappa = 0.88-1.00) for the remaining features. The size measurements including diameter and volume between ZTE-MRI and CT showed no significant difference (*p* = 0.36 for diameter and 0.60 for volume) and revealed perfect inter-observer (ICC = 0.975–0.980) and inter-modality (ICC = 0.942–0.992) agreements. Multivariable analysis showed that non-smooth margin [odds ratio (OR) = 6.008, *p* = 0.015] was an independent predictor for the significant inter-modality variation of volume.

**Conclusion:**

ZTE lung imaging is feasible as a part of chest MRI in the assessment and surveillance for solid pulmonary nodules or masses larger than 1 cm, presenting perfect agreement with CT in terms of morphologic characterization.

## Introduction

Pulmonary nodules or masses are commonly detected by chest CT in routine clinical practice. Most individuals with pulmonary nodules need regular imaging surveillance for subsequent treatment decisions (1). Likewise, patients with lung malignancies frequently undergo follow-up imaging examinations for the evaluation of treatment response and disease progression or recurrence (2). Chest CT is most generally used as a reference modality to monitor the dynamic change of pulmonary nodules or masses; however, repeated CT examinations increase radiation exposure to patients. MRI is desirably utilized for frequent imaging examinations with the characteristics of non-ionizing radiation and may benefit patients with lung diseases, especially for children and pregnant women. However, the intrinsic physical properties of the lung, e.g., short T2 and low proton density of lung parenchymal tissue, inhomogeneity of magnetic susceptibility at air and soft-tissue interface, and cardiopulmonary motion, hinder the widespread clinical application of lung MRI (3, 4). In addition, relatively long echo times (TEs) in conventional MRI sequences lead to difficulty in capturing rapidly decaying signals of lung tissue and small intrapulmonary structures with very short T2.

Zero echo time (ZTE) imaging is a newly introduced fast gradient echo-based MRI sequence with a minimum susceptibility effect that can overcome the abovementioned challenges (5–7). The features including a three-dimensional (3D) slab excitation and radial center-out acquisition complement the blank of lung MRI by the provision of high-resolution intrapulmonary structural information with a better signal-to-noise ratio (SNR) and contrast-to-noise ratio (CNR) (8, 9). With the capability of capturing the intrinsic signal of the lung parenchyma, it could be a promising lung imaging technique. A recent study reported that lung ZTE imaging can provide additional information in the detection and differentiation of lung lesions and enhance the utility of PET/MRI (10). Thus, this imaging scheme without ionizing radiation is expected to provide enough morphological information for patients with lung nodules who need dynamic surveillance or those with malignant tumors who require follow-up evaluation after treatment. In addition, ultrashort TE (UTE) sequence is another MRI technique that can image tissues with ultrashort T2/T2*, and several studies using UTE have been conducted for the assessment of pulmonary emphysema and various pulmonary parenchyma diseases (11–13). Ohno et al. reported that UTE could provide images that resemble CT with excellent performance in pulmonary nodule detection and evaluation of nodule types (11). Compared with UTE, ZTE shows higher sensitivity to short T2 and greater robustness against eddy currents without required trajectory calibration as well as a shorter scan time and lack of operational noise (14). However, the performance of lung ZTE-MRI in the assessment of pulmonary nodules or masses in comparison with conventional CT in routine practice is still not fully illustrated.

The aim of this study was to evaluate the potential of lung ZTE-MRI in the assessment of solid pulmonary nodules or masses and compare ZTE-MRI with CT in terms of morphologic characterization.

## Materials and Methods

### Patients

This prospective study was approved by our Institutional Review Board (No. YY2021-018) and was conducted in compliance with the Health Insurance Portability and Accountability Act. Written informed consent was obtained from all participants. Between February 2021 and June 2021, 112 adult patients with solid pulmonary nodules or masses larger than 1 cm in diameter detected on chest CT at our institution were enrolled. Patients who did not undergo lung ZTE-MRI scan within 7 days after CT (n = 24) were excluded, those without diagnosis data (n = 11), those without detected definite lesions on ZTE-MRI (n = 5), and those showing apparent differences in lung lesions between ZTE-MRI and CT due to acute inflammatory change (n = 1). After application of the exclusion criteria, 71 patients (52 men and 19 women; age range, 18–83 years; mean age, 61.3 ± 12.0 years) were finally enrolled in this study. The diagnosis of pulmonary lesions was confirmed by histopathology or imaging follow-up by typical imaging features.

### Image Acquisition

Chest CT scanning was implemented using a 16-slice CT system (Lightspeed 16, GE Healthcare) at our institution. All examinations were scanned in a craniocaudal direction, with or without contrast medium. CT images were acquired with the following parameters: tube voltage, 120 kV; tube current, 100–440 mA; rotation time, 0.5 s; pitch, 1.375:1; collimation, 20 mm (16 × 1.25 mm); field of view, 350 × 350 mm; and standard soft-tissue algorithm reconstruction. The images were reconstructed at a section thickness of 2.5-mm increments. The radiation doses [median, interquartile range (IQR)] for volumetric CT dose index (CTDIvol) and dose-length product (DLP) were 9.5 mGy (7.5, 11.2 mGy) and 319.8 mGy · cm (249.4, 418.2 mGy · cm), respectively.

MRI examinations were performed without injection of a gadolinium agent using a commercial 3T scanner (Discovery MR750w, GE Healthcare) with a 36-channel body coil. All patients received respiratory-triggered ZTE lung imaging without fat suppression in a silent scan algorithm to accept end-expiratory images with the following parameters: field of view (isotropic), 320 × 320 mm; frequency, 256; slice thickness, 2.0 mm; number of slices, 120; number of motion-resolved datasets, 1; acquisition resolution, 2.0-mm isovoxel; receiver bandwidth, 41.67 kHz; flip angle, 2°; number of spokes per segment, 256; and scan time (mean, range), 187 s (177–194 s). In addition, single-shot echo-planar diffusion-weighted imaging (DWI) was performed for some patients (n = 57) during free-breathing with the following parameters: field of view, 360 × 360 mm; repetition time, 2,500 ms; echo time, 61.4 ms; slice thickness, 3.6 mm; spacing between slices, 3.6 mm; signal averages, 2; matrix 128 × 96; b values selected, 0, 100, and 800 s/mm^2^.

### Qualitative Evaluation

All images were reviewed and analyzed in the Picture Archiving and Communication System (PACS) at our institution. Radiologists 1 and 2 (with 11 years and 18 years of experience in chest imaging, respectively) independently evaluated ZTE-MRI and CT images of all patients. There was a 2-week interval between the evaluation of ZTE-MRI and CT. A third senior radiologist with 25 years’ experience in chest imaging was consulted if there was a disagreement in results, and the senior reader’s decision was finally adopted. For the qualitative evaluation of image quality, the overall diagnostic acceptability of ZTE-MRI and CT was scored using a five-point scale according to the criteria used by Bae et al., respectively, based on the visualization of intrapulmonary vessels and bronchi, and sharpness of diaphragmatic contour as well as noise and artifacts (15).

For the evaluation of imaging features on ZTE-MRI and CT, the following data were recorded (16): 1) site of lesion, indicated as right upper lobe (RUL), right middle lobe (RML), right lower lobe (RLL), left upper lobe (LUL), or left lower lobe (LLL). If a patient had multiple lesions, then the largest one was selected for assessment; 2) location, indicated as central or peripheral; 3) shape, indicated as complex, round, or oval; 4) margin, indicated as smooth or non-smooth; 5) cavitation; 6) air bronchogram; 7) thickening of the adjacent pleura (including the pleural fissures); 8) satellite nodules in primary tumor lobe; 9) nodules in non-tumor lobes; 10) pleural retraction; 11) emphysema; 12) fibrosis; 13) pleural attachment; 14) attachment to vessel; and 15) pleural effusion.

### Quantitative Evaluation

For the quantitative evaluation of image quality, signal intensities (SIs) of the lung parenchyma, pulmonary lesions, and normal structures on axial images of ZTE-MRI were measured. Radiologist 1 drew circular regions of interest (ROIs) in the lung parenchyma, pulmonary nodules or masses, tracheal lumen, tracheal wall, peripheral bronchus, peripheral pulmonary vessel, and aorta according to the previously described methods (15). All measurements were performed three times, and the mean values were recorded and used in this study. The size of the ROI was fitted to the diameter of the structure, and for the heterogeneous pulmonary lesions, only the solid components without cavitation were analyzed. The SNR was calculated as the mean SI of target structure/noise. The CNR of intrapulmonary structures was calculated as [mean SI_structure_ − mean SI _lung_]/noise. The noise was defined as the standard deviation (SD) of the SI in the tracheal lumen.

For the evaluation of unidimensional diameter and 3D volume of the pulmonary lesions on ZTE-MRI and CT, radiologist 1 measured the maximum diameter of the lesions on the axial images and manually delineated the lesion edges slice by slice using ITK-SNAP software (www.itksnap.org, v. 3.6.0), and the 3D volume of interest (VOI) reconstruction was performed to generate the lesion volume (17). There was also a 2-week interval between the evaluation of ZTE-MRI and CT. Radiologist 2 repeated the unidimensional and 3D measurements on ZTE-MRI and CT in randomly selected 50 patients following the above process.

### Statistical Analysis

Continuous variables were expressed as mean ± SD or median with IQR, and categorical variables were expressed as number (percentage). Image quality scores between ZTE-MRI and CT were compared using the Wilcoxon signed-rank test. The inter-modality agreement of imaging features was determined by the percent of concordant cases and the kappa value. Intraclass correlation coefficient (ICC) was used to assess inter-modality and inter-observer agreement for the diameter and volume of the pulmonary lesions. The diameter or volume measurements between ZTE-MRI and CT were also compared using the Wilcoxon signed-rank test, Pearson’s correlation analysis, and Bland–Altman analysis. ICC or the kappa value was interpreted as follows: 0.20 or less, poor; 0.21–0.40, fair; 0.41–0.60, moderate; 0.61–0.80, substantial; and 0.81 or greater, almost perfect agreement. Univariable and multivariable logistic regression analyses with backward stepwise selection were performed to identify the potential factors associated with significant variation (> 10%) of volume between ZTE-MRI and CT. Statistical analyses were performed using IBM SPSS statistics software (version 25.0, SPSS Inc., Chicago, IL, USA). *P*-values less than 0.05 were considered statistically significant.

## Results

Patient characteristics of the study population are summarized in **Table 1**. The diagnosis of the pulmonary nodules (n = 31) or masses (n = 40) was as follows: lung cancer, 54; metastasis, 3; tuberculoma, 6; abscess, 2; and inflammatory nodule, 6. The site of the lesions was as follows: RUL, 16; RML, 3; RLL, 15; LUL, 20; and LLL, 17. All lung ZTE-MRI examinations were successfully performed without any adverse events.

**Table 1:** Patient characteristics of the study population.

	N (%)
No.	71 (100)
Age (years)	61.3 ± 12.0
Sex (male/female)	52 (73.2)/19 (26.8)
Smoking history	40 (56.3)
Diagnosis	
Lung cancer	54 (76.1)
Metastasis	3 (4.2)
Tuberculoma	6 (8.5)
Abscess	2 (2.8)
Inflammatory nodule	6 (8.5)
TNM stage (I/II/III/IV)^*^	13 (24.1)/11 (20.4)/16 (29.6)/14 (25.9)
Site of lesion	
RUL	16 (22.5)
RML	3 (4.2)
RLL	15 (21.1)
LUL	20 (28.3)
LLL	17 (23.9)
Lesion diameter	
1–2 cm	10 (14.1)
2–3 cm	21 (29.6)
3–4 cm	12 (16.9)
4–5 cm	9 (12.7)
>5 cm	19 (26.7)

LLL, left lower lobe; LUL, left upper lobe; RLL, right lower lobe; RML, right middle lobe; RUL, right upper lobe.

^*^TNM stage is available for cases with lung cancer (n = 54).

### Assessment of Image Quality

**Figure 1:**
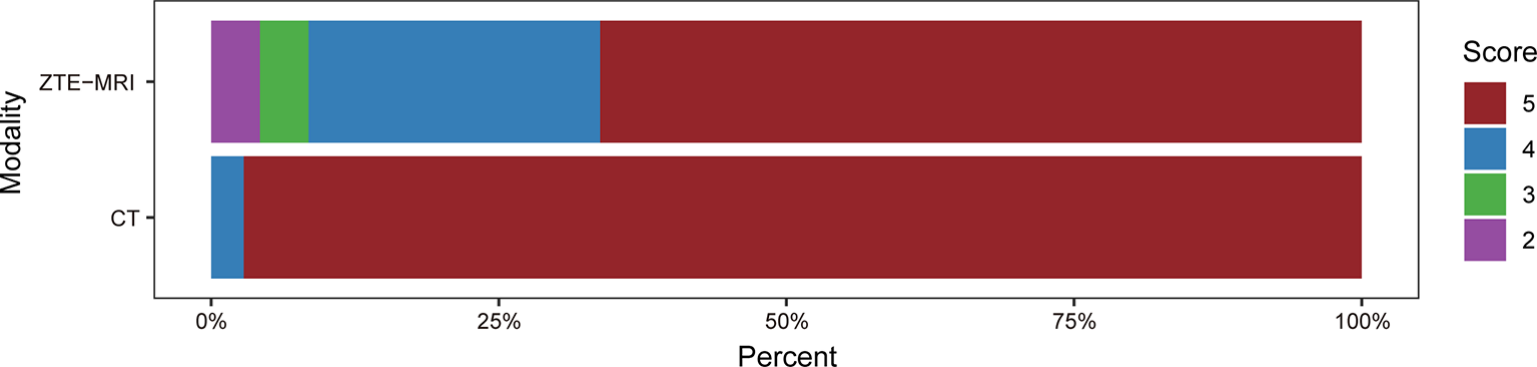
Histogram of the subjective image quality scores for lung ZTE-MRI and CT.

**Figure 2:**
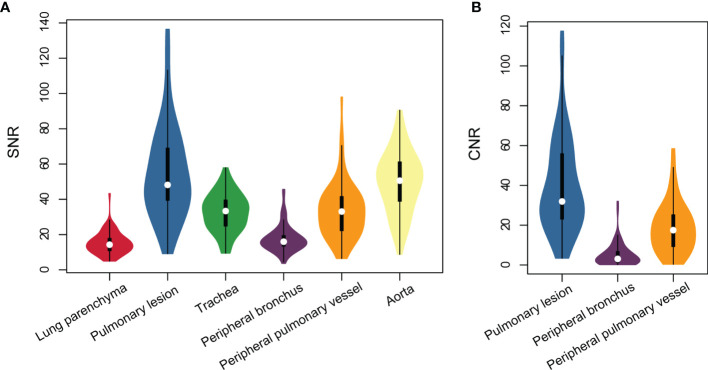
Violin plots represent SNR of all structures **(A)** and CNR of intrapulmonary structures **(B)** on lung ZTE-MRI.

The subjective image quality score in terms of overall acceptability for intrapulmonary vessels and bronchi was lower in ZTE-MRI (4.54 ± 0.77 vs. 4.97 ± 0.17; *p* < 0.001) than in CT (**Figure 1**). The SNRs of the lung parenchyma, pulmonary nodules or masses, trachea, peripheral bronchus, peripheral pulmonary vessel, and aorta, and the CNRs of the pulmonary nodules or masses, peripheral bronchus, and peripheral pulmonary vessel are presented in **Figures 2A** **, B**.

### Inter-Modality Agreement of Imaging Features

**Figure 3:**
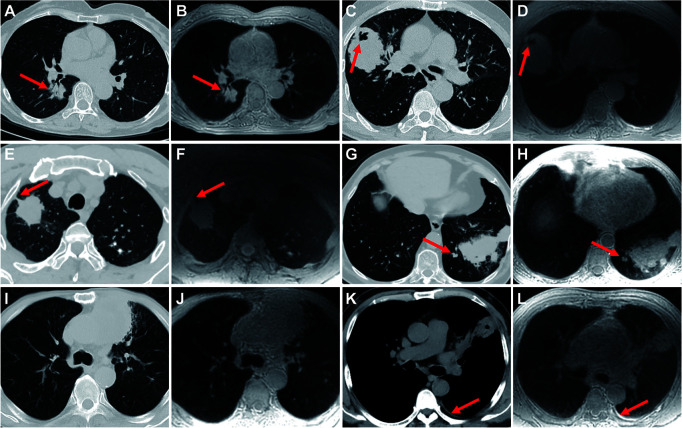
Representative images show the imaging features on CT and ZTE-MRI. Lung adenocarcinoma in the right lower lobe on CT **(A)** and ZTE-MRI **(B)**, showing complex shape and air bronchogram sign; lung adenocarcinoma in the right middle lobe on CT **(C)** and ZTE-MRI **(D)**, showing non-smooth margin and cavitation; lung adenocarcinoma in the right upper lobe on CT **(E)** and ZTE-MRI **(F)**, showing pleural retraction; lung squamous carcinoma in the left lower lobe on CT **(G)** and ZTE-MRI **(H)**, showing satellite nodules in the primary tumor lobe and pleural attachment; lung adenocarcinoma carcinoma in the left upper lobe on CT **(I)** and ZTE-MRI **(J)**, showing emphysema; lung abscess in the left upper lobe on CT **(K)** and ZTE-MRI **(L)**, showing pleural effusion in small amounts.

Inter-modality agreement between ZTE-MRI and CT for the imaging features was moderate for emphysema (kappa = 0.50), substantial for fibrosis (kappa = 0.76), and almost perfect (kappa = 0.88–1.00) for the remaining features, as shown in **Table 2**. The representative cases of CT and ZTE-MRI for characterization of the imaging features are provided in **Figures 3A** **–L**.

**Table 2:** Inter-modality agreement for imaging features between ZTE-MRI and CT.

Imaging features	ZTE-MRI	CT	N (% of concordance)	kappa
Location			71/71 (100)	1.00
Central	17 (23.9)	17 (23.9)		
Peripheral	54 (76.1)	54 (76.1)		
Shape			71/71 (100)	1.00
Complex	35 (49.3)	35 (49.3)		
Round or oval	36 (50.7)	36 (50.7)		
Margin			69/71 (97.2)	0.92
Smooth	18 (25.4)	16 (22.5)		
Non-smooth	53 (74.6)	55 (77.5)		
Cavitation	11 (15.5)	12 (16.9)	70/71 (98.6)	0.95
Air bronchogram	4 (5.6)	5 (7.0)	70/71 (98.6)	0.88
Thickening of the adjacent pleura	40 (56.3)	40 (56.3)	71/71 (100)	1.00
Satellite nodules in primary tumor lobe	26 (36.6)	27 (38.0)	70/71 (98.6)	0.97
Nodules in non-tumor lobes	20 (28.2)	21 (29.6)	70/71 (98.6)	0.97
Pleural retraction	25 (35.2)	25 (35.2)	71/71 (100)	1.00
Emphysema	12 (16.9)	27 (38.0)	56/71 (78.9)	0.50
Fibrosis	22 (31.0)	30 (42.3)	63/71 (88.7)	0.76
Pleural attachment	36 (50.7)	36 (50.7)	71/71 (100)	1.00
Attachment to vessel	9 (12.7)	10 (14.1)	70/71 (98.6)	0.94
Pleural effusion	10 (14.1)	10 (14.1)	71/71 (100)	1.00

ZTE, zero echo time.

### Inter-Modality and Inter-Observer Agreement of Size Measurements

**Figure 4:**
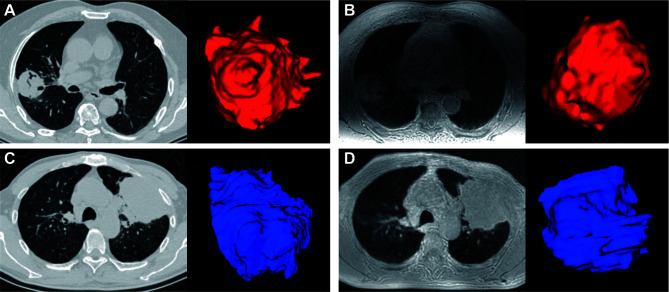
Manual segmentation of pulmonary lesions (red or blue) using volume rendering. CT **(A)** and ZTE-MRI **(B)** in the axial view showed that the measured volumes of a lung tumor in the right upper lobe were 40.7 and 46.6 mm^3^, respectively; CT **(C)** and ZTE-MRI **(D)** in the axial view show that the measured volumes of another lung tumor in the left upper lobe were 138.8 and 145.8 mm^3^, respectively.

**Figure 5:**
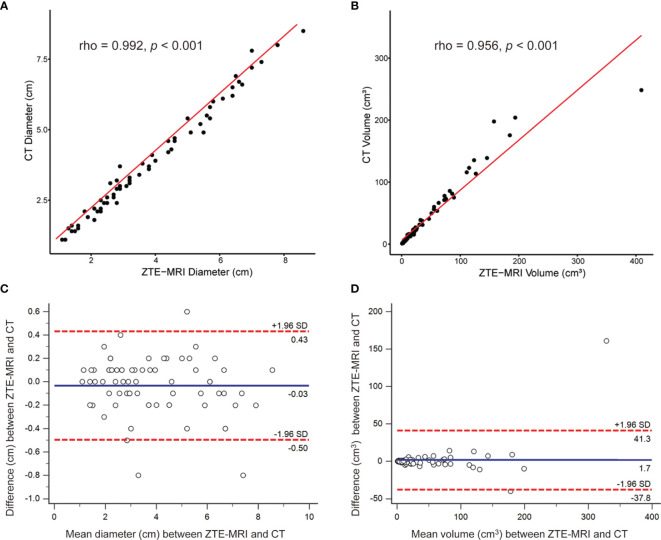
Inter-modality correlation and agreement for lesion measurements. Scatter plots demonstrat the correlations between ZTE-MRI and CT for diameter **(A)** and volume **(B)**. Bland–Altman plots show the inter-modality agreement between ZTE-MRI and CT for diameter **(C)** and volume **(D)**.

Manual 3D segmentation of lung lesions was performed for all patients (**Figures 4A** **–D**). The 71 pulmonary nodules or masses had a median diameter of 3.2 cm (IQR: 2.3, 5.5 cm) on ZTE-MRI and 3.3 cm (IQR: 2.2, 5.4 cm) on CT, and the corresponding volume was 15.1 cm^3^ (IQR: 6.0, 55.4 cm^3^) on ZTE-MRI and 16.1 cm^3^ (IQR: 6.8, 56.1 cm^3^) on CT (**Table 3**). The average differences in the measured diameter or volume between ZTE-MRI and CT were small, and no significant difference was found (*p* = 0.36 for diameter and *p* = 0.60 for volume). The inter-modality ICC value was 0.992 [95% confidence interval (CI): 0.988, 0.995] for diameter and 0.942 (95% CI: 0.909, 0.961) for volume, respectively. Scatter plots (**Figures 5A, B**) demonstrate the strong correlations between ZTE-MRI and CT for diameter (rho = 0.992, *p* < 0.001) and volume (rho = 0.956, *p* < 0.001) measurements. Bland–Altman plots (**Figures 5C, D**) show the inter-modality agreements between ZTE-MRI and CT, with the means and limits of agreement (LoA) being −0.03 cm (−0.50, 0.43) for diameter and 1.7 cm^3^ (−37.8, 41.3) for volume.

**Table 3:** Inter-modality agreement for size measurements between ZTE-MRI and CT.

Measurements	ZTE-MRI	CT	*p*	ICC (95% CI)	Pearson’s r
Diameter (cm)	3.2 (2.3, 5.5)	3.3 (2.2, 5.4)	0.36	0.992 (0.988, 0.995)	0.992
Volume (cm^3^)	15.1 (6.0, 55.4)	16.1 (6.8, 56.1)	0.60	0.942 (0.909, 0.961)	0.956

CI, confidence interval; ICC, intraclass correlation coefficient; ZTE, zero echo time.

**Figure 6:**
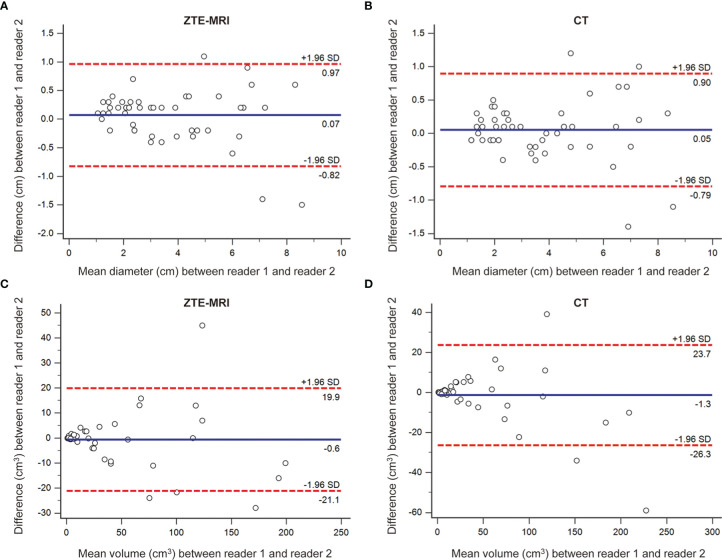
Inter-observer agreement for lesion measurements on ZTE-MRI and CT. Bland–Altman plots show the agreements of diameter on ZTE-MRI **(A)** and CT **(B)**, and the agreements of volume on ZTE-MRI **(C)** and CT **(D)**.

The results of inter-observer agreement of lesion measurements for ZTE-MRI and CT are presented in **Table 4** and demonstrated in the Bland–Altman plots (**Figures 6A** **–D**), which shows that the mean and LoA were 0.07 cm (−0.82, 0.97) on ZTE-MRI and 0.05 cm (−0.79, 0.90) on CT for diameter and −0.6 cm^3^ (−21.1, 19.9) on ZTE-MRI and −1.3 cm^3^ (−26.3, 23.7) on CT for volume. Diameter and volume measurements of different raters, on both ZTE-MRI and CT, were significantly inter-correlated (ICC = 0.975-0.980).

**Table 4:** Inter-observer agreement for size measurements between ZTE-MRI and CT.

Measurements	Reader 1	Reader 2	ICC (95% CI)
ZTE-MRI			
Diameter (cm)	3.0 (2.2, 5.6)	3.1 (2.0, 5.2)	0.976 (0.958, 0.986)
Volume (cm^3^)	12.2 (4.4, 57.4)	10.7 (3.8, 56.9)	0.980 (0.966, 0.989)
CT			
Diameter (cm)	3.2 (2.1, 5.4)	3.5 (1.9, 5.0)	0.979 (0.964, 0.988)
Volume (cm^3^)	13.0 (4.1, 61.6)	12.0 (4.3, 55.7)	0.975 (0.956, 0.986)

CI, confidence interval; ICC, intraclass correlation coefficient; ZTE, zero echo time.

### Factors Associated With Significant Inter-Modality Variation of Volume

**Figure 7:**
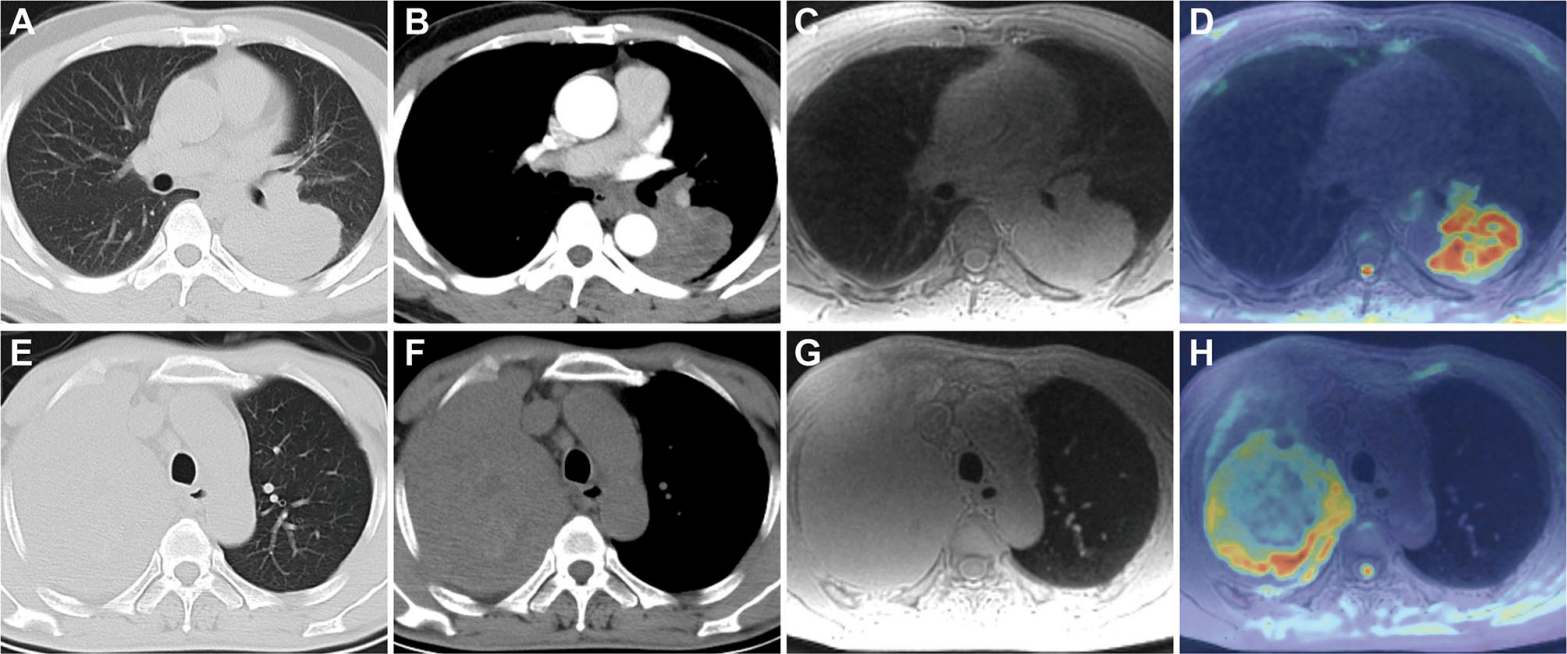
Lung CT and MRI images of two patients without or with significant inter-modality variation of volume. A 48-year-old man with lung adenocarcinoma in the left lower lobe on axial CT lung window **(A)**, mediastinal window **(B)**, and ZTE-MRI **(C)**; ADC map **(D)** showed the consistent lesion extent with ZTE-MRI and CT; a 59-year-old man with lung adenocarcinoma in the right upper lobe on axial CT lung window **(E)**, mediastinal window **(F)**, and ZTE-MRI **(G)**; ADC map **(H)** shows the actual lesion extent which was inconsistent with ZTE-MRI; it is confusing to distinguish the boundary between the non-smooth lesion and surrounding lung tissue with obstructive atelectasis on ZTE-MRI.

To further explore the potential factors associated with the significant inter-modality variation of volume, univariable and multivariable logistic regression analyses were performed (**Table 5**). The results showed that non-smooth margin [odds ratio (OR) 6.008, 95% CI 1.419, 25.444; *p* = 0.015], emphysema (OR 0.198, 95% CI 0.059, 0.664; *p* = 0.009), and pleural attachment (OR 0.219, 95% CI 0.072, 0.662; *p* = 0.007) were independent predictors for the significant inter-modality variation of volume. **Figures 7A** **–H** show two representative cases without or with significant inter-modality variation of volume.

**Table 5:** Factors associated with significant inter-modality variation of volume.

Variables	Univariable OR (95% CI)	*p*-value	Multivariable OR (95% CI)	*p*-value
Age (per year increase)	0.995 (0.957–1.035)	0.805		
Male gender	1.111 (0.388–3.181)	0.844		
Malignant lesion	1.381 (0.425–4.490)	0.592		
Lesion diameter (per cm increase)	0.977 (0.952–1.002)	0.075	–	–
Location (central vs. peripheral)	0.470 (0.152–1.455)	0.190		
Shape (complex vs. round or oval)	1.867 (0.728–4.788)	0.194		
Margin (non-smooth vs. smooth)	2.640 (0.809–8.616)	0.108	6.008 (1.419–25.444)	0.015
Cavitation	1.550 (0.441–5.444)	0.494		
Air bronchogram	1.594 (0.250–10.168)	0.622		
Thickening of the adjacent pleura	0.674 (0.263–1.729)	0.412		
Pleural retraction	1.950 (0.725–5.248)	0.186		
Emphysema	0.346 (0.127–0.942)	0.038	0.198 (0.059–0.664)	0.009
Pleural attachment	0.261 (0.098–0.698)	0.007	0.219 (0.072–0.662)	0.007

CI, confidence interval; OR, odds ratio; ZTE, zero echo time.

## Discussion

In this study, we assessed the performance of lung ZTE-MRI by prospectively head-to-head comparison with CT in the assessment of solid pulmonary nodules or masses (over half of the cohort), including image quality, imaging features, and size measurements. The results showed that the image quality of ZTE-MRI was slightly inferior to CT, but the overall display of intrapulmonary structures and lesions on ZTE-MRI in clinical practice was acceptable. The imaging features on ZTE-MRI showed good consistency with CT except for emphysema and fibrosis, especially for the relationship between pulmonary lesions and pleura. Furthermore, ZTE-MRI revealed perfect inter-modality agreement with CT and inter-observer agreement for the diameter and volume measurements.

ZTE imaging is a 3D slab excitated gradient echo-based sequence and radial center-out filling technique. It has certain restrictions concerning image geometry and scan time but offers flexibly multiple views and permits post-processed averaging through slices of arbitrary orientation and thickness, which improves SNR after selecting the anatomy of interest while maintaining full in-plane resolution (18, 19). Our results indicated that CT was superior to ZTE-MRI in subjective evaluation of image quality such as the more clear depiction of normal structures. Considering that MRI has a lower spatial resolution than CT and is sensitive to magnetic susceptibility, lung MRI is still challenging regarding the delineation of fine intrapulmonary structures, but the overall display of pulmonary nodules was acceptable for the purpose of clinical use. Measured SNR and CNR of intrapulmonary structures on ZTE-MRI images were also in line with a previous report (15). Despite that the ZTE-MRI images were acquired in a quiet and free-breath mode using respiratory triggering, long-time scanning may induce respiratory motion artifacts in some patients, especially for those with severe emphysema or poor lung function (20, 21). ZTE-MRI image collection was performed within a limited time window around end-expiration because patients’ breathing pattern is most consistent and the breathing time is longer in this phase (22). Despite the use of gating, image blurring occurred routinely, especially near the apex pulmonis and diaphragm. In future research, retrospective soft gating techniques would be tried to improve the image quality of ZTE-MRI.

Imaging features on ZTE-MRI showed good consistency with CT except for emphysema and fibrosis. However, some previous reports showed that ZTE-MRI or UTE-MRI were useful to accurately and reliably detect the pathomorphological alterations and quantify the volumetric extent of emphysematous lungs (12, 20, 23). Further studies with quantitative analysis are needed to clarify this controversy. Notably, the nodule-pleura interface margin can be depicted by ZTE-MRI. An earlier study has also shown that MRI offers superior contrast between pleura and adjacent tissues compared with CT (24). Hence, head-to-head comparison in our study suggested that ZTE-MRI may be served as an important approach for the assessment of pleural invasion of pulmonary nodules or masses. The clinical utility of ZTE-MRI for the detection of pleural invasion in patients with lung malignancies, particularly the Pancoast–Tobias tumors, deserves to be verified (25).

Size evaluation of pulmonary nodules or masses is crucial for pathological characterization and therapy monitoring. The evaluation of lesion size varying with time during the follow-up period is the cornerstone for tumor treatment response in the conventional Response Evaluation Criteria in Solid Tumors (RECIST) (26, 27). In our study, ZTE-MRI showed perfect inter-modality agreement with CT and inter-observed agreement for the diameter and volume measurements. A previous UTE-MRI study showed systematically underestimated diameter measurements of pulmonary nodules by approximately 1–2 mm compared with CT, which was in line with our results and possibly explained by smooth structure margins on MRI and blurry lesion margins due to free-breathing image acquisition condition (12). Although diameter measure has been commonly used in clinical practice in the symbol of growth or dynamic change of lesions, volume measure is increasingly recommended in the provision of supplementary information for assessing treatment response and predicting survival in lung cancer patients, particularly those with large, irregular lesions (28, 29). Previous studies indicated that there was very good concordance of lung nodule volumes between CT and UTE-MRI, in both phantom and human settings (30). Tsim et al. reported that MRI volumetry gained superiority over CT for malignant pleural mesothelioma in terms of the association with patient survival (24). Thus, high-resolution ZTE-MRI should be useful for the size evaluation including diameter and volume of pulmonary nodules or masses, which has the potential to obviate frequent chest CT scans, especially in patients who are vulnerable to radiation exposure. Besides, non-smooth margin was found to be an independent predictor for the significant inter-modality variation of volume, which may be caused by the distinct delineation of the peripheral part of the irregular lesions and the adjacent lung tissue on the two imaging modalities (31). In our cohort, there was an eccentric case with non-smooth margin tumor that showed significantly larger volume on ZTE-MRI compared with CT (shown in **Figures 7C, D**), resulting in the outlier in the inter-modality agreement of volume measurement.

This study had several limitations. First, a relatively small sample size was used in our study. Second, only patients with solid pulmonary nodules or masses larger than 1 cm were enrolled. Our study was primarily designed to evaluate the potential of ZTE-MRI lung imaging in comparison with CT for lesion morphologic characterization, instead of detection rate. To further validate the performance of ZTE-MRI in the assessment of sub-solid or ground glass opacification nodules and smaller nodules, future research with larger sample size, specific populations (such as pregnant women), and multiple lesion types are needed. Third, CT images obtained with breath-holding in deep inspiration whereas ZTE-MRI images acquired with respiratory gating under free-breathing on a different date brought possible discrepancies between CT and ZTE-MRI. Finally, readers analyzed the lung ZTE-MRI images and reference standard CT images, which could introduce bias in the evaluation of the image quality, imaging features, and size measurements. To minimize this bias, readers conducted the analyses of ZTE-MRI images at a 2-week interval from the analyses of CT images.

## Conclusion

ZTE lung imaging was feasible as a part of chest MRI without contrast agent in the assessment and surveillance of solid pulmonary nodules or masses larger than 1 cm, presenting perfect agreement with CT in terms of morphologic characterization.

## Data Availability Statement

The raw data supporting the conclusions of this article will be made available by the authors, without undue reservation.

## Author Contributions

QL, ZF, and WZ were involved in the design of the work, analysis of data, and approval of the final version to be published. QL, ZF, and WL contributed to the statistical analysis of the data and drafting the manuscript. WF, LH, XC, and ZM contributed to the acquisition and analysis of data. All authors contributed to the article and approved the submitted version.

## Funding

This study was supported by the Innovation Guidance Project of Clinical Medical Technology in Hunan Province, China (2020SK52704, QL) and the Natural Science Foundation of Hunan Province, China (2021JJ30695, WZ).

## Conflict of Interest

The authors of this manuscript declare a relationship with GE Healthcare. WL is a senior scientist in GE Healthcare (Beijing, China) and assisted the ZTE sequence setting and necessary training for this study. She has no intention to apply for a patent based on this paper or invent any product and did not provide any financial support.

The remaining authors declare that the research was conducted in the absence of any commercial or financial relationships that could be construed as a potential conflict of interest.

## Publisher’s Note

All claims expressed in this article are solely those of the authors and do not necessarily represent those of their affiliated organizations, or those of the publisher, the editors and the reviewers. Any product that may be evaluated in this article, or claim that may be made by its manufacturer, is not guaranteed or endorsed by the publisher.
